# Barrett’s Epithelium to Esophageal Adenocarcinoma: Is There a “Point of No Return”?

**DOI:** 10.3389/fgene.2021.706706

**Published:** 2021-09-17

**Authors:** Anshuman Panda, Mi ryung Shin, Christina Cheng, Manisha Bajpai

**Affiliations:** ^1^Rutgers Cancer Institute of New Jersey, Rutgers, The State University of New Jersey, New Brunswick, NJ, United States; ^2^Department of Gastroenterology and Hepatology, Rutgers Robert Wood Johnson Medical School, Rutgers, The State University of New Jersey, New Brunswick, NJ, United States

**Keywords:** Barrett’s epithelium carcinogenesis, esophageal adenocarcinoma, cell culture model, gastroesophageal reflux, gene expression patterns

## Abstract

**Background:** Esophageal adenocarcinoma (EA) arises from Barrett’s epithelium (BE), and chronic gastroesophageal reflux disease is considered the strongest risk factor for disease progression. All BE patients undergo acid suppressive therapy, surveillance, and BE removal by surgery or endoscopic ablation, yet the incidence of EAC continues to increase. Despite the known side effects and mortality, the one-size-fits-all approach is the standard clinical management as there are no reliable methods for risk stratification.

**Methods:** Paired-end Illumina NextSeq500 RNA sequencing was performed on total RNA extracted from 20-week intervals (0, 20, 40, and 60 W) of an *in vitro* BE carcinogenesis (BEC) model to construct time series global gene expression patterns (GEPs). The cells from two strategic time points (20 and 40 W) based on the GEPs were grown for another 20 weeks, with and without further acid and bile salt (ABS) stimulation, and the recurrent neoplastic cell phenotypes were evaluated.

**Results:** Hierarchical clustering of 866 genes with ≥ twofold change in transcript levels across the four time points revealed maximum variation between the BEC20W and BEC40W cells. Enrichment analysis confirmed that the genes altered ≥ twofold during this window period associated with carcinogenesis and malignancy. Intriguingly, the BEC20W cells required further ABS exposure to gain neoplastic changes, but the BEC40W cells progressed to malignant transformation after 20 weeks even in the absence of additional ABS.

**Discussion:** The transcriptomic gene expression patterns in the BEC model demonstrate evidence of a clear threshold in the progression of BE to malignancy. Catastrophic transcriptomic changes during a window period culminate in the commitment of the BE cells to a “point of no return,” and removal of ABS is not effective in preventing their malignant transformation. Discerning this “point of no return” during BE surveillance by tracking the GEPs has the potential to evaluate risk of BE progression and enable personalized clinical management.

## Introduction

Esophageal adenocarcinoma (EA) is a morbid cancer with less than 15% 5-year survival (Eloubeidi et al., 2003; Polednak, 2003). Over the last few decades, the EA incidence rate has increased more than any other type of cancer in the United States, Europe, and Asia (Fernandes et al., 2006; Shibata et al., 2008; Pohl et al., 2010; Edgren et al., 2013; Hur et al., 2013). Flawed anatomical and physiological conditions in the patients (Coppola and Karl, 1999; Brown et al., 2008; Jung, 2011) can lead to chronic reflux of acidic bile from the stomach into the esophagus, a condition called gastroesophageal reflux disease (GERD) (Runge et al., 2015). GERD is strongly implicated in the development of specialized columnar metaplasia or Barrett’s epithelium (BE) at the junction of the distal esophagus and the stomach (Lagergren et al., 1999; Hofstetter et al., 2001). This specialized tissue, BE, is a known precursor of EA, posing a 30- to 120-fold higher risk compared with the non-BE population (Wild and Hardie, 2003). A recent retrospective study that explored differences between progressors and non-progressors in a large cohort of 460 patients previously diagnosed with BE and followed for over a 21-year period confirmed the presence of LGD as a risk factor for EAC (Kambhampati et al., 2020). The risk is known to escalate as metaplasia progresses from low-grade dysplasia (LGD) to high-grade dysplasia (HGD), although there exists substantial evidence that most patients with BE never progress to EA (Conio et al., 2003; Sharma et al., 2006), and only 0.5–1% of patients with BE develop EA every year (Thomas et al., 2007; Yousef et al., 2008; Desai et al., 2012).

Patients with chronic GERD, regardless of their individual risk of disease progression, are prescribed acid-suppressive drugs, BE surveillance (upon confirmation of BE metaplasia), and antireflux surgery (after dysplastic changes are noted) as part of the standard of care in clinical settings. These acid-suppressive approaches are based on claims of regression of BE and reduced risk of development of dysplasia and EA in some cases (Gore et al., 1993; Neumann et al., 1995; Malesci et al., 1996; DeMeester et al., 1998; Low et al., 1999; Peters et al., 1999; Hofstetter et al., 2001; El-Serag et al., 2004; Hillman et al., 2004). However, long-term proton pump inhibitor (PPI) therapy compromises quality of life and leads to multiorgan complications (Ciovica et al., 2006). They are often ineffective in patients with refractory GERD or in patients with an anatomical defect. Antireflux surgery (fundoplication), advocated for dysplastic stages of BE, is also associated with risk of mortality and compromised quality of life (Spechler et al., 2001). Endoscopic methods of thermal and non-thermal BE ablation alongside “antacid therapy” have gained recognition, but the safety and long-term benefits from these procedures remain to be established. Also, recurrence of BE after complete eradication of intestinal metaplasia within 1 year is a critical issue, and there are no definite guidelines for surveillance of patients after these surgical procedures (Komanduri et al., 2018).

Apart from the lack of therapeutic choices to prevent BE progression, the inability to discriminate between progressive and non-progressive dysplastic BE compromises EA risk prediction and complicates disease management. Several groups of investigators have utilized clinical samples from different histological grades of BE to identify stage-specific molecular signatures for predicting risk of BE progression (Brabender et al., 2004; Razvi et al., 2007; Dulak et al., 2013; Varghese et al., 2015). Ironically, no consistent molecular signature/s for BE progression have yet been identified.

This report postulates that there is a distinctive window period of catastrophic changes and commitment to BE carcinogenesis identifiable from global gene expression patterns (GEPs) of biopsy samples collected from multiple time points during EA surveillance that can indicate the course of the disease. Using a mathematical algorithm and RNA-seq datasets from time series samples collected every 20 weeks (0, 20, 40, and 60 W) from a previously described BE carcinogenesis (BEC) model (Das et al., 2011), we demonstrate GEPs coinciding with the progressive neoplastic changes in the non-neoplastic human Barrett’s epithelial cells (BAR-T) due to prolonged intermittent acid and bile salt (ABS) exposure. These GEPs highlight a window period of remarkable changes in expression of genes associated with carcinogenesis between BEC20W and BEC40W time points that lead to commitment of the BEC40W cells to malignant transformation, i.e., “point of no return” without the need for any further ABS stimuli. The ability to discern this window period that propels the cells to the “point of no return” by tracking GEPs during surveillance for EA will add to the current paradigm in stratifying patients who respond to acid suppression from those who may not, and select for those who require, mucosal resection or radiofrequency ablation or carry druggable targets for preventing BE progression to EA.

## Materials and Methods

### Cell Culture and Acidic Bile Salt Exposure

The BE carcinogenesis (BEC) model, described elsewhere (Das et al., 2011), was derived after exposing the human Barrett’s epithelial cell line (BAR-T) to acidified (pH = 4) bile salt GCDC (glycochenodeoxycholic acid, physiological component of gastric refluxate) referred to as acidic bile salt or ABS in text for 5 min every day for 1 year. The human telomerase (h-TERT) immortalized BAR-T cell line (kind gift from Dr. Rhonda Souza, Baylor University Medical Center at Dallas) was established from the biopsies of a patient with non-dysplastic Barrett’s epithelium (Jaiswal et al., 2007). The BEC model cells displayed characteristic change in cell shape and clustering of cells after about 34 weeks of ABS exposure, and malignant characteristics like loss of contact inhibition (foci formation) and ability to grow in soft agar after 58 weeks and more of ABS exposure, as described earlier (Das et al., 2011). The cells were frozen away in liquid nitrogen at regular intervals and have been found to retain the characteristic cellular properties like altered shape, clustering of cells, foci, and soft agar colony formation after revival from liquid nitrogen storage (Bajpai et al., 2012).

For the observations presented in this report, the BEC20W and BEC40W cells were revived from liquid nitrogen storage and split into two sets each. These time points were selected due to their characteristic cellular phenotypes change in cell shape and clustering of cells, loss of contact inhibition, ability to grow in soft agar reported earlier (Das et al., 2011). One set of cells from each time point was exposed to ABS for another 20 weeks (BEC20W + 20 weeks with ABS and BEC40W + 20 weeks with ABS), and the other set was maintained in parallel in cell culture conditions without any further ABS exposure (BEC20W + 20 weeks without ABS and BEC40W + 20 weeks without ABS). This was done to confirm the cellular changes previously observed in the BEC model, and those characteristics were used to evaluate the need for ABS exposure during the critical window period (between BEC20W and BEC40W). Therefore, the BEC20W + 20 weeks with ABS is a replicate of BEC40W, and BEC40W + 20 weeks with ABS is a replicate of BEC60W. All cell lines used in this experiment tested negative for mycoplasma contamination using the Mycoplasma PCR detection kit (Sigma).

### Cell Shape and Microscopy

The cellular characteristics described above were the endpoints to assess the neoplastic progression in BEC20W and BEC40W cells in the presence or absence of further ABS exposure for another 20 weeks. A total of 0.02 × 10^6^ cells were plated into each well of a six-well plate to observe the cell shape at low cell density, using an Olympus CK40 microscope at × 45 magnification.

### Colony Formation in Soft Agar

For evaluation of malignant phenotype, 6,000 cells were plated per well in a six-well plate in growth medium containing 0.4% agar (500 μl per well); the base agar layer had growth medium containing 0.8% agar (1 ml per well). Four weeks after plating, the colonies were stained (cell transformation detection assay, Chemicon) and counted. Two-sided two-sample *t*-test was performed to compare three replicates each of the control and ABS-exposed groups, and statistical significance was assessed at *p* < 0.05.

### Foci Formation Assay

The cells were plated at a low density of 5 × 10^5^ per 100-mm cell culture dish and were then allowed to grow for 4–5 weeks and beyond confluency. For assessment of distribution patterns of the cells, these plates were fixed with 10% methanol, 10% acetic acid solution, and stained with 20% ethanol, 0.4% crystal violet for 5 min and recorded using the Olympus CK40 microscope at × 10 magnification. ImageJ^1^ was used to calculate the percentage of surface area on the plates occupied by the cells (foci). Two-sided two-sample *t*-test was performed to compare three replicates each of the control and ABS exposed groups, and statistical significance was assessed at *p* < 0.05.

### RNA-Seq and Enrichment Analysis

Paired-end Illumina NextSeq500 sequencing was performed on total RNA extracted from BEC0W, BEC20W, BEC40W, and BEC60W cells (the read count data is available in Supplementary Table 1). Of the 18,560 genes for which read count data were available, 5,684 genes had <5 reads in every sample and were therefore excluded. Read count data for the remaining 12,876 genes, with a minimum of five reads in at least one sample, was normalized using the “rlog” function of the “DESeq2” package (Love et al., 2014). The “pheatmap” package in R was used for hierarchical clustering, and ToppGene suite (Chen et al., 2009) was used for enrichment analysis. The ToppGene suite also includes mouse genome informatics (MGI) that curates and infers phenotypic similarity between mouse models and human diseases based on reports published in literature.

### Classification of Genes Based on Their Expression Pattern in the Time Series

In case of genes with ≥ twofold expression variation across the four time points, expression of a gene at each time point was considered high or low (H/L) depending on whether it was higher/lower than the average expression of the same gene across the four time points, i.e., BEC0W, BEC20W, BEC40W, and BEC60W, respectively. Each gene was placed in one of the 14 categories (2^4^–2, since HHHH and LLLL are excluded) based on whether it had high/low expression at the four time points (BEC0W, BEC20W, BEC40W, and BEC60W, respectively) of the series.

### Confirmatory Quantitative Real-Time PCR

To validate RNA-seq results, select genes were confirmed by qRT-PCR on the Bio-Rad CFX96 instrument using the Quantitech SYBR green PCR kit (Qiagen) to follow the same trend, and the list of primers is available in Supplementary Table 2. One-sided Wilcoxon rank sum test was used to compare the normalized qRT-PCR data at different time points, and statistical significance was assessed at *p* < 0.05.

## Results

### Transcriptomic Changes in the Barrett’s Epithelial Carcinogenesis Model

#### The Distinctive Time Series Gene Expression Patterns

**FIGURE 1:**
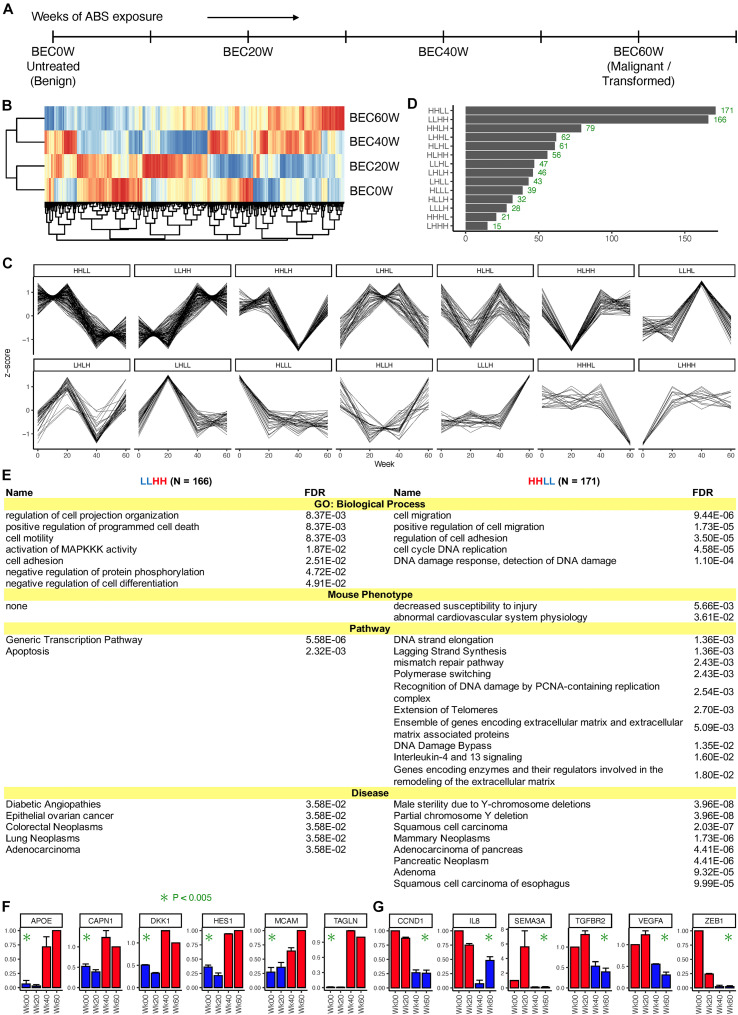
Overall pattern of time series changes in gene expression. **(A)** The outline of the BEC model and time points selected for transcriptomic analyses. **(B)** Heatmap (with hierarchical clustering) of the expression data of 866 genes that showed a twofold or higher variation in expression across the four time points. **(C)** Classification of the genes based on whether the expression at the four time points (BEC0W, BEC20W, BEC40W, and BEC60W) are higher or lower (H/L) than the average of the four time points. **(D)** Number of genes in each category. **(E)** Biological processes, mouse phenotypes, pathways, and diseases enriched in the genes of the LLHH (left) and HHLL (right) patterns. **(F,G)** Confirmatory quantitative PCR for randomly selected genes from LLHH and HHLL patterns.

Paired end RNA-sequencing was performed on the BEC0W cells (not exposed to ABS), and three serial time points, BEC20W, BEC40W, and BEC60W (Figure 1A). In the normalized RNA-seq data, expression of most genes varied less than twofold across the four time points, and only 866 genes showed a twofold or higher variation in expression across the four time points. Figure 1B shows a heat map (with hierarchical clustering) of the expression data of these 866 genes, where the BEC20W sample clustered with the BEC0W sample; the BEC40W sample clustered with the BEC60W sample. This suggests that the transcriptome of BEC20W is more similar to BEC0W cells and that of the BEC40W is more similar to BEC60W cells, and the major transcriptomic changes in the BEC model occurred in the window period between the BEC20W and BEC40W.

The 866 genes with ≥ twofold expression variation across the four time points were further classified based on their time-series expression pattern into 14 categories (Figure 1C). Two categories had disproportionately high number of genes (Figure 1C) – HHLL, 171 genes that have high expression in BEC0W and BEC20W but low expression at BEC40W and BEC60W and LLHH, 166 genes that have low expression at BEC0W and BEC20W but high expression at BEC40W and BEC60W. These genes are listed in Supplementary Table 3, and the number of genes in the remaining categories are shown in Figure 1D.

Figure 1E lists the biological processes, mouse phenotypes, pathways, and diseases enriched in the genes of the LLHH (left) and HHLL (right) categories. Genes in the LLHH category were associated with cell motility, cell adhesion, cell differentiation, generic transcriptional pathways, and with apoptosis-related pathways, whereas genes associated with cell migration, cell adhesion, cell cycle, DNA damage response and extracellular matrix repair were in the HHLL category. Both categories of genes are known to be associated with multiple types of cancer, particularly adenocarcinomas and GI cancers (pancreatic, colorectal, and esophageal). Gene expression pattern of some genes from LLHH (*APOE*, *CAPN1*, *DKK1*, *HES1*, *MCAM*, and *TAGLN*) and HHLL (*CCND1*, *IL8*, *SEMA3A*, *TGFBR2*, *VEGFA*, and *ZEB1*) categories were randomly selected and validated using qRT-PCR (Figures 1F,G, respectively).

#### Transcriptomic Changes in Specific Window Periods

**FIGURE 2:**
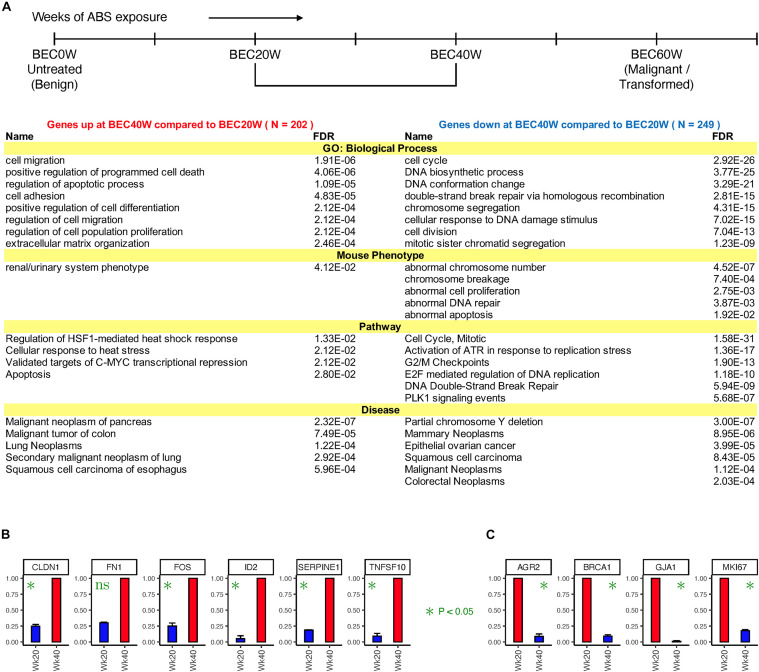
Time series changes in gene expression from BEC20W to BEC40W. **(A)** Biological processes, mouse phenotypes, pathways, and diseases enriched in the genes that went up (left) or down (right) twofold or more from BEC20W to BEC40W. **(B,C)** Confirmatory quantitative PCR for randomly selected genes with increased transcript levels or reduced transcript levels, respectively, between BEC20W and BEC40W.

Expression of 451 genes changed twofold or more from BEC20W to BEC40W, irrespective of their expression at BEC0W or BEC60W time points (listed in Supplementary Table 4). Figure 2A, shows the result of ToppGene enrichment analysis of the 202 genes with increased expression levels (left) and 249 genes with reduced expression levels (right), and these reflect the impairment of similar biological processes and pathways as LLHH and HHLL genes (Figure 1E). The transcript levels of some genes that increased (CLDN1, FN1, FOS, ID2, SERPINEE1, TNFSF10) or decreased (AGR2, BRCA1, GJA1, MIK67) during the window period of BEC20W and BEC40W, was confirmed by qRT-PCR (Figures 2B,C, respectively).

Results of similar enrichment analysis of genes whose expression changed twofold or more from BEC0W to BEC20W (Supplementary Table 5) and BEC40W to BEC60W (Supplementary Table 6), including their biological functions and associated mouse phenotypes and human diseases, along with qRT-PCR confirmation of select genes are shown in Supplementary Figures 1, 2, respectively. The genes that increased in expression between BEC0W to BEC20W are known to participate in biological response to acid injury and various kinds of cellular stress and metabolic activities (Supplementary Figure 1), while those genes that were reduced in transcript levels were involved with the integrity of the extracellular matrix and basement membrane biogenesis and assembly (Supplementary Figure 1). The intuitive model predicted similar gene expression alteration associated with abnormalities in the epidermal–dermal junction morphology and the abdominal wall morphology in the mouse models (Supplementary Figure 1). Similarly, the genes with increased transcript levels between BEC40W to BEC60W were associated with cell cycle and DNA repair (Supplementary Figure 2), while the genes with reduced transcript levels were associated with synthesis of bile acids and bile salts via 24- and 27-hydroxycholesterol (Supplementary Figure 2).

### The “Point of No Return” in the Barrett’s Epithelial Carcinogenesis Model

#### The BEC20W Cells Require Further Acidic Bile Salt Exposure for Progression to Neoplastic Phenotype

**FIGURE 3:**
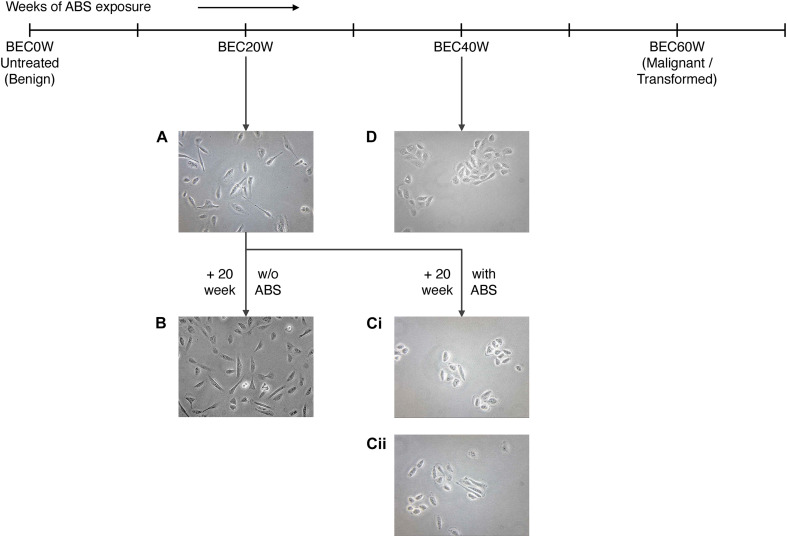
Morphological changes in BEC20W cells further exposed to acid and bile salt (ABS) for 20 weeks: **(A)** BEC20W cells **(B)** BEC20W + 20 weeks cells not exposed to ABS retain their elongated morphology and disperse evenly on the surface of the cell culture plates. **(C)** BEC20W + 20 weeks cells further exposed to ABS for another 20 weeks transform into oblong morphology and exhibit increased cell–cell cohesion **(Ci,ii)** similar to the BEC40W cells **(D)**.

Cell shapes of BEC20W cells (Figure 3A), BEC20W + 20 weeks without ABS (Figure 3B), BEC20W + 20 weeks with ABS (Figure 3C), and BEC40W cells (Figure 3D) was performed under the microscope after plating at low density. The set of BEC20W cells growing for 20 weeks but not exposed to ABS any further (Figure 3B) remained elongated or spindle shaped and evenly distributed on culture plates similar to their parent BEC20W cells (Figure 3A). The set of BEC20W cells further exposed to ABS for an additional 20 weeks displayed change in shape from elongated to oval/circular and distinct clustering of six to eight cells when plated at low density (Figures 3Ci,ii), and these changes appeared as early as 14 weeks (BEC20W + 14 weeks with ABS). This characteristic shape of the cells was the same as presented by the BEC40W cells (Figure 3D) and not their parent BEC20W (Figure 3A).

#### The BEC40W Cells Do Not Require Further Acidic Bile Salt Exposure for Progression to Malignant Phenotype

**FIGURE 4:**
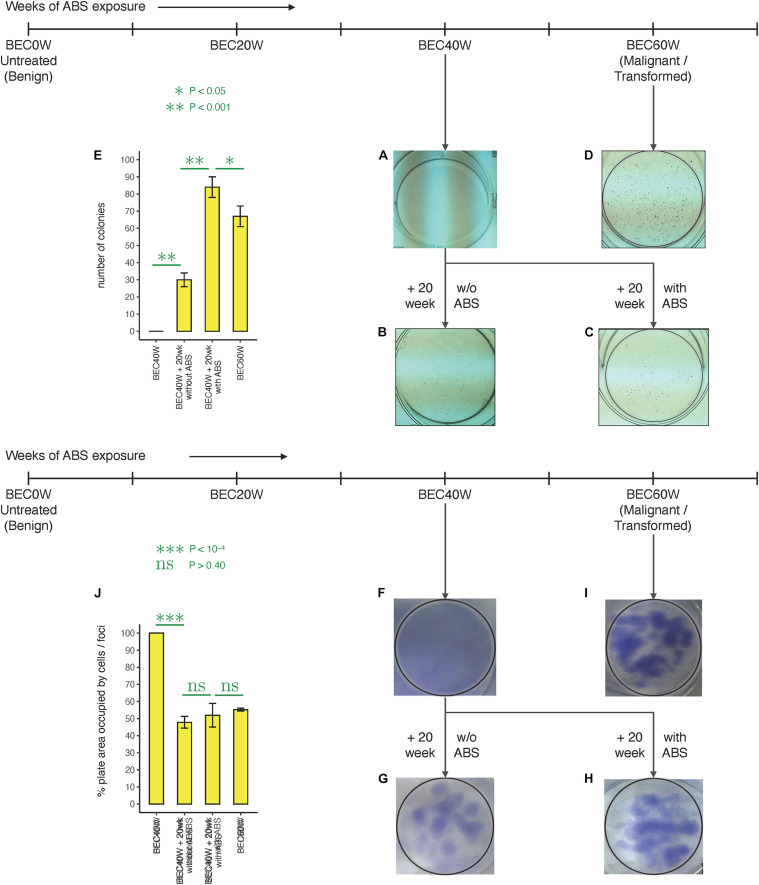
Colony formation in different stages of BEC model: **(A)** The BEC40W cells do not initially form colonies in soft agar. **(B)** BEC40W + 20 weeks begin to form distinct colonies demonstrating loss of substrate adherence dependence after 20 weeks even in the absence of any further ABS. **(C)** The BEC40W + 20 weeks further exposed to ABS form more colonies comparable with the BEC60W cells **(D)** that retain their ability to form colonies even in the absence of stimulation after thawed from storage (representative pictures). **(E)** Total number of colonies compared between the BEC40W + 20weeks with the ABS and without ABS groups, and the BEC60W cells. *Foci formation in different stages of BEC model*: **(F)** The BEC40W cells do not initially form foci when confluent. **(G)** BEC40W + 20 weeks cells begin to form distinct foci demonstrating loss of contact inhibition after 20 more weeks even in the absence of any further ABS **(H)**. The BEC40W + 20 weeks further exposed to ABS form colonies as expected comparable with BEC60W cells **(I)** that retain their ability to form foci even in the absence of stimulation after thawed from storage (representative pictures). **(J)** Percent plate surface occupied by the foci (dark staining patches of multilayered cells) was compared with the monolayer of cells (BEC40W) covering the entire plate (100%).

When BEC40W cells, BEC40W + 20 weeks without ABS, BEC40W + 20 weeks with ABS, and BEC60W cells were compared for properties of malignant transformation, namely, the ability to form soft agar colonies (Figures 4A–E) and the ability to form foci (Figures 4F–I), it was observed that the BEC40W cells did not form soft agar colonies (Figure 4A) or foci (Figure 4F) after revival from storage. However, they acquired the ability to form soft agar colonies (Figure 4B) due to loss of attachment to substrate and foci (Figure 4G) due to loss of contact inhibition after growing for 20 weeks (approximately 20 passages) even without any further ABS exposure. The total area occupied by the multilayered foci (patchy dark staining) on BEC40W + 20 weeks without ABS, BEC40W + 20 weeks with ABS, and BEC60W cells (Figures 4G–I) were compared with the BEC40W cells (Figure 4F) that had a monolayer of cells distributed evenly across the entire plate (even light staining). Although the foci in each of the experimental conditions (Figures 4G–I) occupied about 50% of the plate surface (*p* < 0.4); this was significantly less (*p* < 10^–4^) compared with the untreated BEC40W (Figure 4J). It was also notable that the BEC40W cells exposed to ABS for 20 more weeks (Figure 4C) formed significantly more colonies (Figure 4E) compared with the BEC40W cells growing for another 20 weeks without further ABS exposure (Figure 4B). These observations confirmed that while BEC40W cells are not transformed *per se*, they are already committed to transformation and require only time (not further ABS exposure) to undergo malignant transformation. It was clear that the BEC60W cells retained their ability to form colonies in soft agar (Figure 4D) and foci (Figure 4I) after revival from liquid nitrogen storage. The BEC60W cells continued to form soft agar colonies when allowed to grow for 20 more weeks with or without further ABS exposure (data not shown).

## Discussion

The development of Barrett’s epithelium at the junction of esophagus and stomach due to scarring from acid reflux and its propensity to develop into adenocarcinoma of the esophagus are well established. Acid-suppressive therapy during the early stages of BE or ablative surgery and acid-suppressive therapy at advanced stages are the only recommendations for medical management of BE, although their efficacy in preventing EA remains questionable. Acid-suppressive therapy and anti-reflux surgery reduce exposure of the esophagus to acidic contents of the stomach (Gore et al., 1993; Peters et al., 1999; Weinstein, 2002; El-Serag et al., 2004; Gashi et al., 2018; Tan et al., 2018). Although, in most cases, the change in length of the BE segment is reportedly uncommon, the acid suppression prevents disease progression during these early stages of BE. Although the length of the BE segment is directly related to the risk for dysplasia, histological determination of dysplastic changes calls for more aggressive disease management. Once low-grade dysplasia (LGD) is identified in BE, maximal acid suppression, close surveillance, and evaluation for endoscopic resection or ablation procedures are recommended (Shaheen et al., 2016). A set of reliable biomarkers that could identify patients at higher risk of EA remains elusive; and regardless of the advancement in endoscopic procedures for removing dysplastic BE, lack of objective biomarkers continues to compromise risk prediction and, hence, the value of surveillance for EAC. The need to identify patients at higher risk for esophageal adenocarcinoma has prompted several investigations into identifying a “gene signature” for high-risk BE dysplasia. Several animal and cell culture models have been developed and utilized to understand the molecular mechanisms of BE progression, although each has its own limitations in emulating the BE disease process (Bus et al., 2012; Garman et al., 2012). Several scrupulous investigations involving cutting edge technology and massive numbers of clinical tissues from different histological grades of BE, collected prospectively over several years, have been utilized to identify the gene signature for BE progression (Brabender et al., 2004; Razvi et al., 2007; Dulak et al., 2013; Varghese et al., 2015) that could discriminate progressors from non-progressors and/or predict risk for BE progression. The investigations have now turned to next-generation sequencing to analyze the wholesome genomic alterations during BE progression, and efforts are also underway to include the epigenome, transcriptome, and proteome data (Contino et al., 2017).

It is intriguing that the observed changes induced by acid and bile exposure in the BEC model were irreversible, but not necessarily oncogenic in the earlier stages, and progressive transformation of BEC20W cells (not committed to malignant transformation) into altered shape and adhesion/clustered behavior could be prevented simply by removing further ABS trigger. This was suggestive of success with acid-suppressive strategies before the “point of no return.” The ABS removal strategy did not succeed with the BEC40W cells as they showed characteristics of malignant transformation (foci and soft agar colony formation) even in the absence of further ABS exposure, although their ability to form colonies was not as pronounced as the BEC40W cells further exposed to ABS for 20 weeks or the BEC60W cells. This observation highlighted the significance of the “point of no return” strongly associated with catastrophic changes in gene expression between the BEC20W and BEC40W and commitment of cell to malignant transformation even in the absence of ABS exposure. Although we have previously reported the widespread genetic and epigenetic changes after 20 weeks of ABS exposure (Bajpai et al., 2013), the clustering of BEC0W and BEC20W together as one set, and BEC40W and BEC60W as another, confirmed the magnitude of changes that occur between 20 and 40 weeks. The committed BEC40W cells had altered cell shape and cell adhesion properties, increased basal proliferation potential, and increased resistance to cell death in response to further ABS exposure (Das et al., 2011). ToppGene enrichment analyses of the genes altered during the specific intervals combined with MGI confirmed alteration of biological functions associated with the phenotypes displayed during the same interval. Our previous observations (Bajpai et al., 2012, 2021) also point to chromosomal rearrangements during this critical period in the BEC model that may account for catastrophic changes propelling the commitment of BE cells to malignant progression. There are other pieces of evidence in literature supporting the presence of chromosomal aberrations and stage-specific gene expression signatures associated with disease progression from BE to EAC (Garewal et al., 1989; Barrett et al., 1996; Newell et al., 2019). However, those are accumulative data and still inconclusive for replication in clinical care.

The major limitation of this study is its reliance on a simplistic *in vitro* cell culture model. However, similar acid and bile exposure induced increase in cell proliferation (Hong et al., 2010a), DNA damage, and resistance to apoptosis (Huo et al., 2011), perhaps via induction of the NFkB pathway (Hormi-Carver et al., 2009), oxidative damage (Hong et al., 2010b), and induction of reactive oxygen species leading to DNA double-strand breaks (Zhang et al., 2009) in the BAR-T cells, have been reported by other investigators. Change in appearance of cells from elongated to circular/oval shape has also been observed in normal esophageal cell lines after long-term acid and bile exposure (Minacapelli et al., 2017). Increased cell–cell adhesion and loss of attachment to substrate was noted in the BEC model (Das et al., 2011), and induction of epithelial mesenchymal characteristics (altered adhesion to substrate and increased motility) was confirmed in the BEC20W cells after further acid and bile exposure, via activation of the VEGF pathway (Zhang et al., 2019). It is also notable that the distinctive phenotypic characteristics and transcriptomic changes acquired after chronic ABS exposure to the BAR-T cells were retained by the non-neoplastic (BEC20W) as well as the neoplastic (BEC40W) and malignantly transformed (BEC60W) BEC cells even after storage in liquid nitrogen and subsequent retrieval and represent a viable mechanism of BE carcinogenesis. The distinguishing phenotypic endpoints make this *in vitro* model a promising tool for mechanistic studies to understand the function of biological molecules implicated in clinical BE progression.

In conclusion, we postulate the presence of emergent gene expression patterns that signify time series changes during the progression of BE to EA and provide evidence for a presumable “point of no return” in Barrett’s carcinogenesis, beyond which irrevocable changes commit the BE cells to malignant phenotype that may not be rescued by acid and bile suppression (therapy). Discerning this “point of no return” using serial biopsies of patients undergoing surveillance has the potential to find molecular identifiers to enable the development of individualized risk prediction model and personalized clinical management of BE patients.

## Data Availability Statement

The original contributions presented in the study are included in the article/Supplementary Material, further inquiries can be directed to the corresponding author.

## Author Contributions

AP developed the algorithm used in this manuscript, performed the interpretation of gene expression data and all computational analyses, and wrote and revised the manuscript. MS and CC performed cell culture-based experiments, gene expression studies, and acquisition of data. MB led the conception and design, cell culture experiments, analysis, interpretation, and compilation of data, and writing of the manuscript. All authors approved the final version of the manuscript to be published.

## Conflict of Interest

The authors declare that the research was conducted in the absence of any commercial or financial relationships that could be construed as a potential conflict of interest. The handling editor declared a past co-authorship with one of the authors AP.

## Publisher’s Note

All claims expressed in this article are solely those of the authors and do not necessarily represent those of their affiliated organizations, or those of the publisher, the editors and the reviewers. Any product that may be evaluated in this article, or claim that may be made by its manufacturer, is not guaranteed or endorsed by the publisher.

## Footnotes

1
http://imagej.nih.gov/ij

## Supplementary Material

Supplementary Figure 1 **(A)** Time-series changes in gene expression from BEC0W to BEC20W: Biological processes, mouse phenotypes, pathways, and diseases enriched in the genes that went up (left) or down (right) 2 fold or more from BEC0W to BEC20W. **(B,C)** Confirmatory quantitative PCR for randomly selected genes with increased transcript levels or reduced transcript levels, respectively, between BEC0W and BEC20W.

Supplementary Figure 2 **(A)** Time-series changes in gene expression from BEC40W to BEC60W: Biological processes, mouse phenotypes, pathways, and diseases enriched in the genes that went up (left) or down (right) 2 fold or more from BEC40W to BEC60W. **(B,C)** Confirmatory quantitative PCR for randomly selected genes with increased transcript levels or reduced transcript levels, respectively, between BEC40W and BEC60W.

Supplementary Table 1 Total FFKM read count data for the four time points of the BEC model (0, 20, 40, and 60 W).

Supplementary Table 2 List of primers used for confirmatory qPCRs.

Supplementary Table 3 List of genes in LLHH and HHLL categories.

Supplementary Table 4 List of genes up/down two fold or more in BEC40W compared to BEC20W and their log_2_ Fold Changes.

Supplementary Table 5 List of genes up/down two fold or more in BEC20W compared to BEC0W and their log_2_ Fold Changes.

Supplementary Table 6 List of genes up/down two fold or more in BEC60W compared to BEC40W and their log_2_ Fold Changes.
