# Supplementary figures and images for: Barrett’s Epithelium to Esophageal Adenocarcinoma: Is There a “Point of No Return”?
Source: Front Genet. 2021 Sep 17;12:706706. doi: 10.3389/fgene.2021.706706 (PMC8485939; doi:10.3389/fgene.2021.706706)

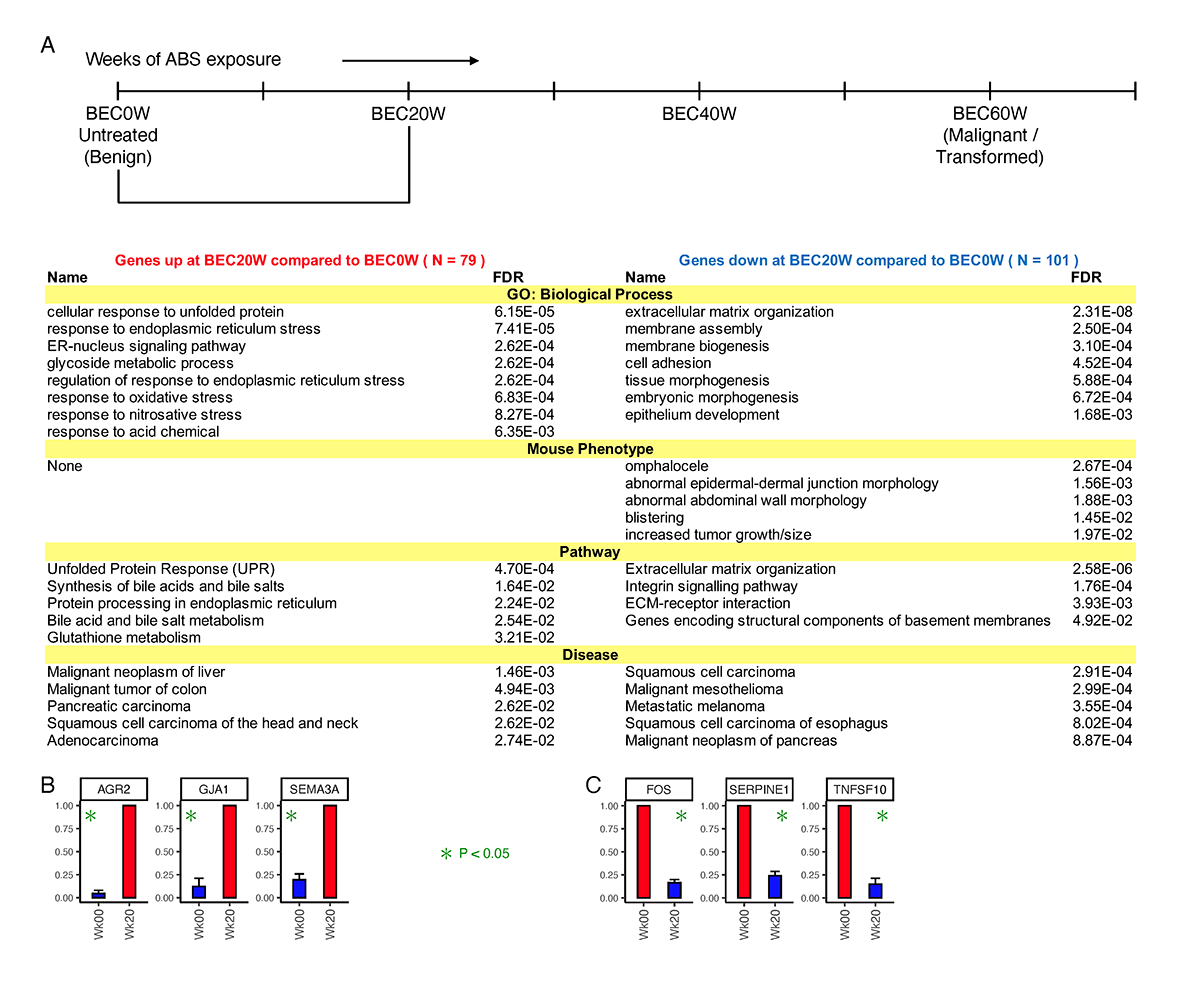

Supplement: Supplementary Figure 1 — (A) Time-series changes in gene expression from BEC0W to BEC20W: Biological processes, mouse phenotypes, pathways, and diseases enriched in the genes that went up (left) or down (right) 2 fold or more from BEC0W to BEC20W. (B,C) Confirmatory quantitative PCR for randomly selected genes with increased transcript levels or reduced transcript levels, respectively, between BEC0W and BEC20W. [file Image_1.TIF]

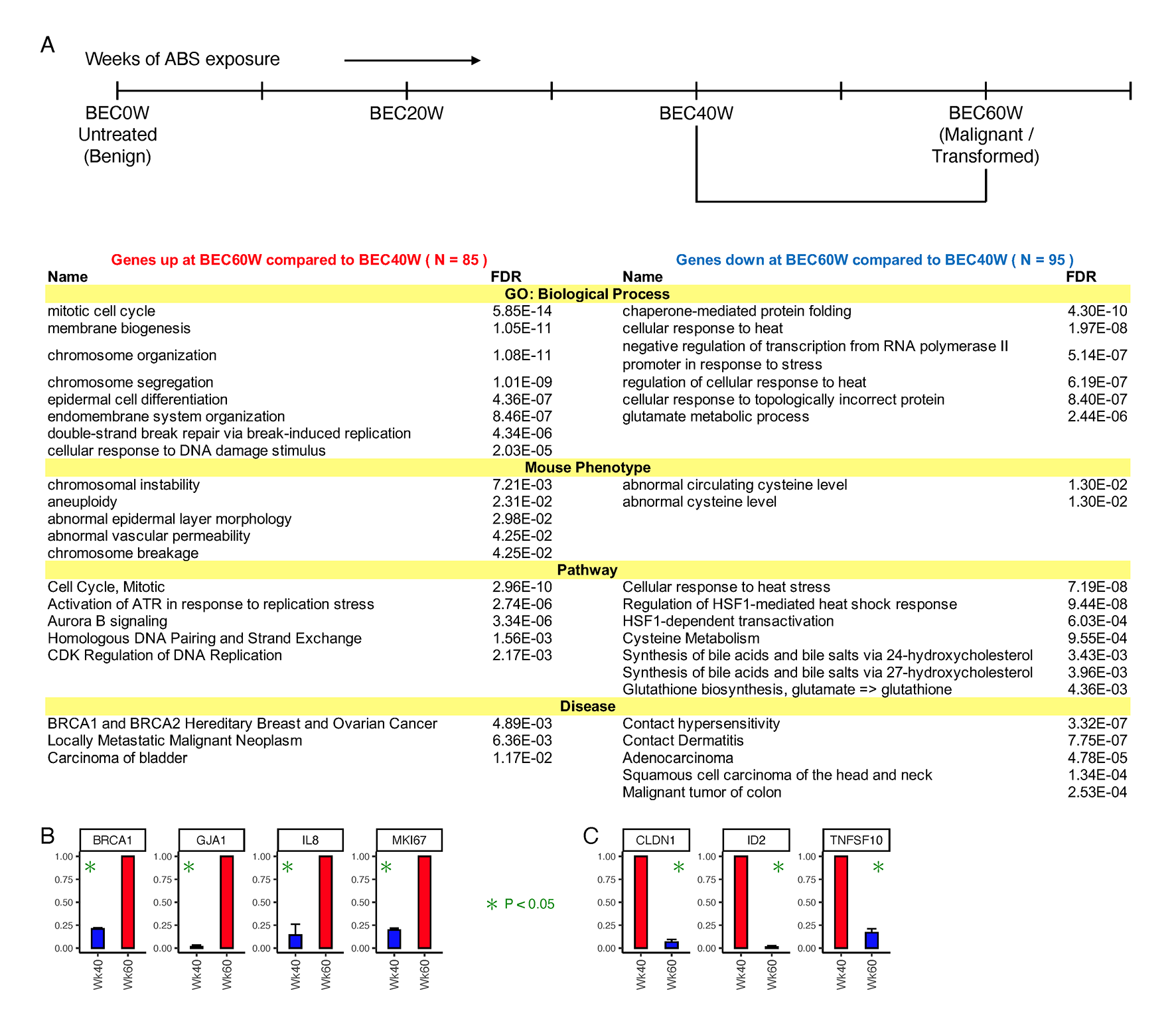

Supplement: Supplementary Figure 2 — (A) Time-series changes in gene expression from BEC40W to BEC60W: Biological processes, mouse phenotypes, pathways, and diseases enriched in the genes that went up (left) or down (right) 2 fold or more from BEC40W to BEC60W. (B,C) Confirmatory quantitative PCR for randomly selected genes with increased transcript levels or reduced transcript levels, respectively, between BEC40W and BEC60W. [file Image_2.TIF]
